# Minimum Information Variability in Linear Langevin Systems via Model Predictive Control

**DOI:** 10.3390/e26040323

**Published:** 2024-04-10

**Authors:** Adrian-Josue Guel-Cortez, Eun-jin Kim, Mohamed W. Mehrez

**Affiliations:** 1 Centre for Fluid and Complex Systems, Coventry University, Priory St, Coventry CV1 5FB, UK; ejk92122@gmail.com; 2 Zebra Technologies, 2100 Meadowvale Blvd, Mississauga, ON L5N 7J9, Canada; m.mehrez.said@gmail.com

**Keywords:** information theory, model predictive control, Langevin equations, fluctuations, entropy

## Abstract

Controlling the time evolution of a probability distribution that describes the dynamics of a given complex system is a challenging problem. Achieving success in this endeavour will benefit multiple practical scenarios, e.g., controlling mesoscopic systems. Here, we propose a control approach blending the model predictive control technique with insights from information geometry theory. Focusing on linear Langevin systems, we use model predictive control online optimisation capabilities to determine the system inputs that minimise deviations from the geodesic of the information length over time, ensuring dynamics with minimum “geometric information variability”. We validate our methodology through numerical experimentation on the Ornstein–Uhlenbeck process and Kramers equation, demonstrating its feasibility. Furthermore, in the context of the Ornstein–Uhlenbeck process, we analyse the impact on the entropy production and entropy rate, providing a physical understanding of the effects of minimum information variability control.

## 1. Introduction

Time-varying probability density functions (PDFs) are a preferred approach for characterising the dynamics of various complex systems. PDFs commonly feature in emergent fields, such as active inference [1] or stochastic thermodynamics [2], where their value is derived either through data analysis or by solving the Fokker–Planck (FP) equation associated with an Itô/Stratonovich stochastic differential equation.

Drawing upon control theory [3], when the dynamics of the system under consideration are governed by an FP equation, we can explore control objectives such as the regulation (setting to a constant value) or tracking (following a time-varying reference) of the systems’ time-varying PDFs [4]. While the notion of controlling PDFs may initially appear impractical using conventional control engineering methods, advancements in technologies like optical tweezers have rendered it feasible, which is particularly evident in applications such as colloidal systems [5,6,7,8], with specific implications in biomolecule evolution control [9].

Regarding the control of FP equations, seminal works [10,11] present methodologies to control the system PDF governed by FP equations [12]. Building upon this foundation, ref. [13] discusses a bilinear optimal control problem where the control function depends on time and space. In [14], the authors prove the existence of optimal controls by considering first-order necessary conditions in the optimisation problem.

In applications like Brownian motion, we can control FP equations via reverse-engineering approaches such as the engineered swing equilibration (ESE) method [15,16]. The ESE protocol imposes a solution to the FP equation to obtain the corresponding PDF’s control parameters that provide a shortcut for time-consuming relaxations (for further details, refer to the methods section of [17]). Ref. [8] offers a comprehensive review of similar reverse-engineered approaches.

Since FP equations frequently serve as mathematical descriptions of mesoscopic systems (for further details, see [12]), i.e., systems at the nano/microscale such as molecular motors, the time evolution of the system’s PDF may not only need to adhere to time constraints but also to multiple “thermodynamic” constraints to be deemed “efficient”. For instance, the system may require the minimisation of the entropy production [18,19], a reduction in information variability [20], or an enhancement of self-organisation [21]. The incorporation of these “thermodynamic constraints” into the optimisation process implies an extension of the current literature results on the control of FP equations.

A theory that could offer insights into addressing these optimisation challenges stems from information geometry, a field resulting from the fusion of information theory and differential geometry [22]. As an evolving field, information geometry proposes novel solutions to tasks, such as maximum likelihood estimation [23], state prediction [24,25], the quantification of causality [26,27,28], or maximum work extraction [4,29]. In stochastic thermodynamics [2,30], information geometry aids in obtaining time-varying descriptions of the aforementioned constraints. For instance, based on the well-known Cauchy–Schwartz inequality [31], ref. [32] presents an inequality between the *Fisher divergence* [33] and the *information length* (IL) [25,34] to quantify the disorder in irreversible decay processes. Ref. [35] introduces an inequality describing the information rate as a speed limit on the evolution of any observable. In [4], the geodesic of the IL describes the path with the least statistical variation connecting initial and final probability distributions of the system dynamics (for further details, see [20]). Consequently, information geometry can be employed in a control protocol to enforce geodesic dynamics on the system’s PDF time evolution, achieving the minimum “geometric information variability” [21] and thereby establishing an optimal speed limit for the system’s observables. The primary challenge addressed in this study revolves around devising and applying a technique to achieve this minimum geometric information variability.

Developing an optimal protocol for the time evolution of the system’s PDFs under multiple constraints requires an exploration of existing control algorithms. The literature presents a significant amount of control procedures, spanning from classical PID control [36] to more sophisticated algorithms, like data-driven, model-free, or fractional-order controls (for instance, see [37,38,39]). However, we require an algorithm capable of handling intricate optimisation problems while remaining a feasible option for implementation in future experimental setups. One of the most popular optimisation-based control techniques is the model predictive control (MPC) scheme [40]. Generally, MPC is an online optimisation algorithm designed for constrained control problems, whose advantages have been observed in applications within robotics [41], solar energy [42], or bioengineering [43]. Furthermore, MPC can be easily implemented thanks to packages such as CasADi [44] or the Hybrid Toolbox [45].

Based on the presented discussion, this work offers a solution to an optimisation problem that integrates the concepts of the information length and the quadratic regulator (QR) [46] to guide the system’s PDF time evolution along the path with the minimum geometric information variability (the geodesic of the information length) using MPC. Although we could implement the IL, QR, or MPC in more complex scenarios, we only study their application to stochastic processes described by linear stochastic differential equations. Hence, the system’s PDF will consistently maintain a Gaussian distribution over time, assuming that the system’s initial conditions follow a Gaussian distribution. Constraining the analysis to linear stochastic dynamics allows us to use a set of deterministic differential equations to describe the evolution of the mean and covariance of the Gaussian distribution within the MPC method. The applicability of such a prediction model favours MPC over other alternatives, such as reinforcement learning [47], which could have been explored to determine the geodesic of the information length within a similar context. Additionally, MPC provides a low offline complexity, mature stability–feasibility–robustness theory, and good constraint handling [47].

The algorithm is applied to the Ornstein–Uhlenbeck process [48] and the Kramers equation [25], which both describe a particle over a heath reservoir (*mesoscopic stochastic dynamics*). In practice, changes in temperature and optical tweezers can manipulate the dynamics of both the noise amplitude and mean value in such systems, respectively [2,7,49]. Using previous findings from [50], the effects of the MPC method on the Ornstein–Uhlenbeck process are analysed by comparing IL values with the entropy production and entropy rate in the closed-loop (feedback-controlled) system. As a rigorous closed-loop stability analysis is beyond the scope of this work, we provide only a brief description of the BIBO stability conditions that are considered to constrain the control actions proposed by the MPC. Finally, we give a set of concluding remarks and discuss future work.

To help readers, in the following, we summarise our notations. R is the set of real numbers. x $\in R^{n}$ represents a column vector x of real numbers of dimension *n*, $A\in R^{n\times n}$ represents a real matrix of dimension n×n (bold-face letters are used to represent vectors and matrices), Tr(A) corresponds to the trace of the matrix A, and $A_{ij}$ is the element at row *i* and column *j* of the matrix A. |A|, vec(A), $A^{⊤}$, and $A^{-1}$ are the determinant, vectorisation, transpose, and inverse of matrix A, respectively. The value $I_{n}$ denotes the identity matrix of order *n*. Newton’s notation is used for the partial derivative concerning the variable *t* (i.e., $\frac{\partial y}{\partial t}=\dot{y}$). In addition, the average of a random vector ζ is denoted by μ:=〈ζ〉, with the angular brackets representing the average. Finally, in Table 1, we provide a brief description of the variables used throughout this paper.

## 2. Preliminaries

As mentioned in Section 1, this work considers systems whose dynamics are governed by linear non-autonomous stochastic differential equations described in the form of the following set of Langevin equations:

(1) $$\dot{\zeta }\left(t\right)=A\zeta \left(t\right)+Bu\left(t\right)+\xi \left(t\right),$$

where A and B are n×n and n×1 real time-invariant matrices, respectively; u(t) is a (bounded smooth) external input, and $\xi \in R^{n}$ is a Gaussian stochastic noise given by an *n* dimensional vector of δ-correlated Gaussian noises $\xi_{i}$ (i=1,2,…n), with the following statistical property:

(2) $$\langle \xi_{i}\left(t\right)\rangle =0,\langle \xi_{i}\left(t\right)\xi_{j}\left(t_{1}\right)\rangle =2D_{ij}\left(t\right)\delta \left(t-t_{1}\right),\;D_{ij}\left(t\right)=D_{ji}\left(t\right),\forall i,j=1,\ldots ,n.$$

Readers seeking a comprehensive study of systems governed by Equation (1) can refer to [51]. Additionally, for a concise introduction to linear time-varying and time-invariant systems, ref. [52] provides valuable insights.

The Fokker–Planck equation of (1) can also be utilised to depict the system’s PDF dynamics, characterised by both time *t* and the spatial variable vector $x={\left[x_{1},x_{2},\ldots ,x_{n}\right]}^{⊤}$. This is given as follows [53,54]:

(3) $$\frac{\partial p(x;t)}{\partial t}=-\sum_{i,j=1}^{n}\frac{\partial }{\partial x_{i}}\left[\left(a_{ij}x_{j}+b_{i}u\left(t\right)\right)p\left(x;t\right)\right]+\sum_{i,j=1}^{n}D_{ij}\frac{\partial^{2}}{\partial x_{i}\partial x_{j}}p\left(x;t\right).$$

If the initial PDF is Gaussian, a solution to (3) for p(x;t) at any time *t* is given by [53]

(4) $$p\left(x;t\right)=\frac{1}{\sqrt{\det(2\pi \Sigma )}}e^{-\frac{1}{2}{\left(x-\mu \right)}^{⊤}\Sigma^{-1}\left(x-\mu \right)}.$$

Equation (4) describes the dynamics of the probability distribution of the random variable ζ governed by (1). The value of the mean μ(t) and covariance Σ(t) in a linear stochastic process can be computed from the solution of the following set of differential equations [51]:

(5a) $$\begin{array}{ccc} \dot{\mu }\left(t\right) & = & A\mu (t)+Bu(t), \end{array}$$

(5b) $$\begin{array}{ccc} \dot{\Sigma } & = & A\Sigma \left(t\right)+\Sigma \left(t\right)A^{⊤}+2D\left(t\right), \end{array}$$

where $D\in R^{n\times n}$ is the matrix with elements $D_{ij}\left(t\right)$. In this work, Equations (5a) and (5b) will be used to predict the behaviour of the probability distribution of the random variable x in our control method.

### 2.1. Information Length (IL)

To obtain a minimum geometric information variability in the time evolution of the PDF of Equation (1), we need to investigate the concept of IL in more detail. In mathematical terms, given the joint PDF p(x;t) of the n-th order random variable ζ, we define its IL L as follows:

(6) $$L\left(t\right)=\int_{0}^{t}\left(\sqrt{\int_{R^{n}}p\left(x;\tau \right){\left[\partial_{\tau }\ln p(x;\tau )\right]}^{2}\;d^{n}x}\right)d\tau =\int_{0}^{t}\Gamma \left(\tau \right)\;d\tau .$$

**Figure 1:**
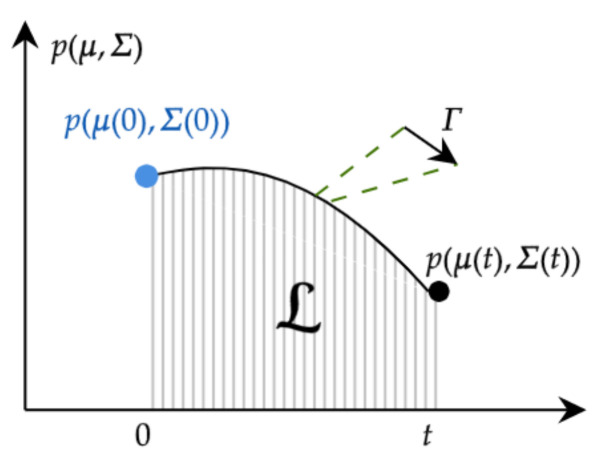
Graphical description of the value of L. From information geometry, L quantifies the number of statistically different states that the system passes through in time from an initial p(x;0) to a final p(x;t).

The value $\Gamma {\left(\tau \right)}^{2}=\int_{R^{n}}p\left(x;\tau \right){\left[\partial_{\tau }\ln p(x;\tau )\right]}^{2}\;d^{n}x$ in (6) corresponds to the square of the Fisher information by considering the time as a parameter, and it is commonly called the information rate [21]. The dimension of 1/Γ≡τ is time, which means that it serves as a dynamical time unit for information change. Hence, as shown in Figure 1, the integration of Γ between time 0 and *t* gives the total information change in that time interval; i.e., L quantifies the number of statistically different states that the system passes through in time from an initial p(x;0) to a final p(x;t) [55]. Furthermore, the IL is a model-free tool that proved to be a true metric between two PDFs in the statistical space [26]. In [33], τ was shown to provide a universal bound on the timescale of transient dynamical fluctuations, independent of the physical constraints on the stochastic dynamics or their function.

For Gaussian distributions, according to [25], we know that Γ can be rewritten in terms of μ and Σ as follows:

(7) $$\Gamma {\left(\tau \right)}^{2}=\dot{\mu }{\left(t_{1}\right)}^{⊤}\Sigma^{-1}\left(t_{1}\right)\dot{\mu }\left(t_{1}\right)+\frac{1}{2}\mathrm{Tr}\left({\left(\Sigma^{-1}\left(t_{1}\right)\dot{\Sigma }\left(t_{1}\right)\right)}^{2}\right).$$

Equation (7) simplifies the analysis of the IL when studying systems like (1).

### 2.2. Information–Thermodynamic Relation

However, while we recognise the utility of the IL as a tool for analysing the dynamics of time-varying PDFs, its physical significance remains unclear. Therefore, establishing a connection to a physical quantity is crucial.

**Figure 2:**
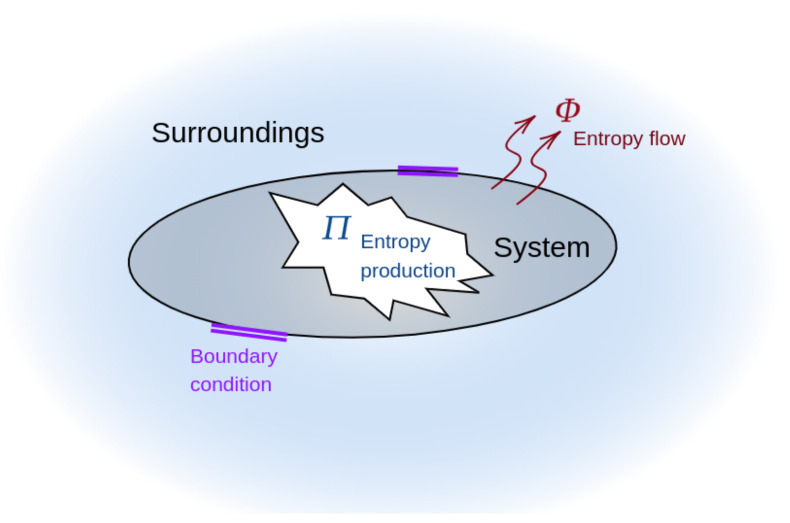
Graphical description of Equation (8). In a closed system, entropy rate $\dot{S}$ corresponds to the subtraction of the entropy produced by the system Π and the entropy exchanged with the environment Φ.

In this context, consider the value of the entropy rate defined as follows: [56]

(8) $$\dot{S}\left(t\right)=-\int_{R^{n}}\dot{p}\left(x;t\right)\ln\left(p(x;t)\right)\;dx=\Pi -\Phi ,$$

where Π is the entropy production due to irreversible processes occurring inside the system and Φ is the entropy flux from the system to the environment. Figure 2 shows a system subject to some boundary conditions to avoid matter exchange (i.e., a closed system). The system produces entropy Π>0∀t and exchanges entropy Φ with the environment (hence, it shares energy). Specifically, Φ>0 (Φ<0) when the entropy flows from the system (environment) to the environment (system). A system with a minimum entropy production Π produces the highest amount of free energy (useful work) [21,57]. We refer the reader to [58,59] for a complete review of thermodynamics. In a first-order linear stochastic differential equation, the value of the information rate Γ can be related to $\dot{S}$ and Π as follows (for further details, see [50]):

(9) $$\Gamma^{2}=\frac{D}{\Sigma }\Pi +{\dot{S}}^{2}\;\forall D,\Sigma >0,$$

where *D* and Σ are the noise amplitude and the variance of the first-order Langevin equation.

Equation (9) can be used to describe the physical effects of a minimum variability control in a dynamical system. For instance, as will be discussed later in Section 4, to obtain a minimum information variability while being out of the equilibrium (i.e., Π>0), the control will exert entropy to the environment, creating a small but negative value for $\dot{S}$. This means that a minimum information variability would not necessarily lead to a maximum free energy but to an optimal path where Π is limited by the value of Γ according to (9). The complete derivation of how to compute the values of $\dot{S},\Pi$, and Φ for a first-order Langevin equation is shown in Appendix C.

### 2.3. Minimum Information Variability Problem

Now that we understand the meaning of the IL, let us explain in more detail how the IL can be used to minimise deviations from the geodesic of the system’s PDF time evolution. In [32], the authors use the inequality $J\left(t_{F}\right)\geq L{\left(t_{F}\right)}^{2}$ where $J=\tau \int_{t_{0}}^{t_{f}}\Gamma^{2}\left(t\right)\;dt=\int_{t_{0}}^{t_{f}}\;dt\int \;dx\frac{1}{p(x;t)}{\left[\frac{\partial p(x;t)}{\partial t}\right]}^{2}$ (Fisher divergence) with $\tau =t_{f}-t_{0}$ and L given by (6). As mentioned in Section 1, such inequality follows from the Cauchy–Schwartz inequality $\int \Gamma^{2}\;dt\int u^{2}\;dt\geq {\left(\int \Gamma u\;dt\right)}^{2}$ with u=1. But, most importantly, the equality holds for the minimum path where Γ is constant (see, e.g., [19,32]), and the deviation from this equality is said to quantify the amount of the disorder in an irreversible process [32].

From [20], such a statement can be clarified by the following procedure. Let us define the time-average for a function f(t) as $E\left[f\left(t\right)\right]=\frac{1}{\tau }\int_{t_{0}}^{t_{f}}f\left(t\right)\;dt$. Then, we can define the time-averaged variance

(10) $$\sigma^{2}=\frac{J-L^{2}}{\tau^{2}}=E\left[\Gamma^{2}\right]-E{\left[\Gamma \right]}^{2}\geq 0.$$

Equation (10) describes an accumulative deviation from the geodesic connecting the initial and final distributions of the system dynamics. Thus, we can conclude that by setting Γ as a constant, we obtain a geodesic that defines a path where the process has the minimum *geometric information variability*.

### 2.4. BIBO Stability of the Linear Stochastic Process

As we address a control problem, it is essential to examine the bounded-input, bounded-output (BIBO) stability of (1). For this purpose, we revisit the BIBO stability conditions for Equations (5a) and (5b), which will be instrumental in our discussion of MPC stability.

**Theorem** **1.** *The mean (* 5a *) and covariance (* 5b *) dynamics of (* 1 *) are BIBO-stable if and only if the eigenvalues $\lambda_{i}$ of the matrix A satisfy the following inequality:*

(11) $$ℜ[\lambda_{i}]<0,$$

*where ℜ[s] stands for the real part of the complex value s∈C.*

**Proof.**  For a detailed proof of this result, please refer to [60,61]. □

**Remark** **1.** *Theorem 1 is considered to be satisfied throughout our examples; i.e., the control method is applied to stable systems only. Furthermore, the control actions are constrained to finite values.*

## 3. Main Results

To guide the system’s PDF time evolution along the geodesic of the IL while achieving a desired set point at time $t=t_{f}$, we propose the following cost function:

(12) $$\;J\;\;=\;\;\int_{0}^{t_{f}}\;\left(I_{L}{\left(\Gamma^{2}\left(\tau \right)\;-\;\Gamma^{2}\left(0\right)\right)}^{2}\;+\;{\left(Y\left(\tau \right)\;-\;Y_{d}\left(\tau \right)\right)}^{⊤}Q\left(Y\left(\tau \right)\;-\;Y_{d}\left(\tau \right)\right)+c^{⊤}\left(\tau \right)Rc\left(\tau \right)\right)d\tau ,$$

where $I_{L}\in R,Q\in R^{\left(n+n^{2}\right)\times \left(n+n^{2}\right)}$; $Y:={\left[\mu ,\mathrm{vec}\left(\Sigma \right)\right]}^{⊤}\in R^{n+n^{2}}$ is the vector of states μ and vec(Σ) that define the time evolution of p(x;t); $Y_{d}={\left[\mu_{d},\mathrm{vec}\left(\Sigma_{d}\right)\right]}^{⊤}$ is the desired position of the $n+n^{2}$ states defined by $\mu_{d}$; and $\Sigma_{d}$ at time *t*, and $c\in R^{m}$ (such that $m\leq 1+n^{2}$) is the vector of controls defined by $c={\left[c_{1},c_{2},\ldots ,c_{m}\right]}^{⊤}:=[u\left(t\right),w:={\left\{\left(D_{ij}|D_{ij}\neq 0\in D\forall i,j=1,2,\ldots ,n\right\}\right]}^{⊤}$. Therefore, $R\in R^{m\times m}$. In this work, we call Equation (12) the *Information Length Quadratic Regulator*(IL-QR). As will be discussed in Section 3, MPC enables us to obtain the solution of (12) via a numerical scheme, circumventing analytic complexities while being applicable to practical scenarios. Analytically finding the geodesic dynamics involves using the solution of the Euler–Lagrange equations of the IL. The steps of such an approach are discussed and successfully applied in [4] for a first-order stochastic differential equation. In Appendix A, we give the details of the procedure when considering a more generalised scenario.

From (12), we observe that the first term on the right-hand side imposes a constant $\Gamma^{2}$ (needed to minimise the deviations from the geodesic [4]). The term involving Q guides the system towards a specified PDF defined by $Y_{d}$. The third term on the right-hand side of (12) regularises the control action c to prevent abrupt changes in the inputs. Furthermore, Q and R are matrices that penalise the error $\epsilon =Y-Y_{d}$ and the control input *u*, respectively. Additionally, $I_{L}$ penalises the square of the error $\left(\Gamma^{2}\left(t\right)-\Gamma^{2}\left(0\right)\right)$, aiming to maintain $\Gamma^{2}\left(t\right)$ close to $\Gamma^{2}\left(0\right)$ over time.

In our approach, the control of the dynamics for μ is governed by u(t), while the dynamics of Σ(t) are adjusted by controlling the noise width using a time-dependent vector w(t), where its elements replace the non-zero constant values of the matrix D in (5b). As discussed in the numerical examples, the noise width can be altered by modifying a macroscopic observable such as temperature (for further details, refer to the Brownian motion models presented in [12]).

### Model Predictive Control

As discussed in Section 1, the solution of our optimisation problem will be computed through the MPC method. Hence, the following discrete optimal control problem encoding the MPC formulation is required:

(13) $$\begin{array}{cc} c=\arg\min_{\tilde{c}} & \left(J_{N}=\sum_{k=0}^{N}\left(I_{L}\;{\left({\hat{\Gamma }}^{2}\left[k\right]-\Gamma^{2}\left[0\right]\right)}^{2}\right.\right.\\ \; & \left.\left.+{\left(\hat{Y}\left[k\right]-Y_{d}\right)}^{⊤}Q\left(\hat{Y}\left[k\right]-Y_{d}\right)+{\tilde{c}}^{⊤}\left[k\right]R\tilde{c}\left[k\right]\right)\right)\\ s.t.\; & {\hat{\Gamma }}^{2}\left[k\right]=f\left(\hat{Y}\left[k\right],\tilde{c}\left[k\right]\right)\\ \; & \hat{Y}\left[k\right]={\left[\mu \left[\tilde{c},k\right],\mathrm{vec}\left(\Sigma \left[\tilde{c},k\right]\right)\right]}^{⊤}\\ \; & \mu \left[\tilde{c},k\right]=A_{d}\mu \left[k-1\right]+B_{d}u\left[\tilde{c},k-1\right]\\ \; & \Sigma \left[\tilde{c},k\right]=A_{d}\Sigma \left[k-1\right]+\Sigma \left[k-1\right]A_{d}^{⊤}+2D\left[\tilde{c},k-1\right]\\ \; & \mu \left[0\right]=m,\Sigma \left[0\right]=S\;\forall m\in R^{n},S\in R^{n\times n}\\ \; & \tilde{c}\left[k\right]={\left[u\left[k\right],w\left[k\right]\right]}^{⊤}\\ \; & c_{l,i}\leq c_{i}\leq c_{u,i}\;c_{l,i},c_{u,i}\in R\forall i=1,2,\ldots ,m. \end{array}$$

In Equation (13), the  ^ symbol over Y and Γ refers to their predicted values over the influence of the control $\tilde{c}$ throughout the optimisation process in the finite horizon of length *N*. Note that the value of $\hat{Y}$ is constrained by the discretised version of the set of Equations (5a) and (5b) given by

(14) $$\begin{array}{ccc} \mu [k] & = & A_{d}\mu \left[k-1\right]+B_{d}u\left[k-1\right], \end{array}$$

(15) $$\begin{array}{ccc} \Sigma [k] & = & A_{d}\Sigma \left[k-1\right]+\Sigma \left[k-1\right]A_{d}^{⊤}+2D\left[k-1\right], \end{array}$$

where $A_{d}=I+T_{s}A$ and $B_{d}=T_{s}B$ if a first-order approximation of the time derivative considering the sample period $T_{s}$ is applied (we apply a fourth-order Runge Kutta instead of a first-order approximation to compute $\hat{Y}$ in our simulations). Note that we have added the argument $\tilde{c}$ in Equation (13) when describing Equations (14) and (15) to emphasise the application of the control during the optimisation procedure. The initial conditions μ[0],Σ[0] of (14) and (15) change every time an optimal control c solution has been computed and they are subject to the measurements m,S of the real/simulated process. Every element $c_{i}$ of the control vector $\tilde{c}$ is constrained by a lower and an upper limit denoted as $c_{l,i}$ and $c_{u,i}$, respectively. Finally, *f* is the function describing the predicted value ${\hat{\Gamma }}^{2}$, which is defined as follows:

(16) $$\begin{array}{c} \;f\left(\hat{Y}\left[k\right],\tilde{c}\left[k\right]\right)={\left(A\mu \left[k\right]+Bu\left[\tilde{c},k\right]\right)}^{⊤}\Sigma {\left[k\right]}^{-1}\left(A\mu \left[k\right]+Bu\left[\tilde{c},k\right]\right)\\ \;\;\;\;\;\;\;\;\;+\frac{1}{2}\mathrm{Tr}\left({\left(\Sigma {\left[k\right]}^{-1}\left(A\Sigma \left[k-1\right]+\Sigma \left[k-1\right]A^{⊤}+2D\left[\tilde{c},k-1\right]\right)\right)}^{2}\right). \end{array}$$

**Figure 3:**
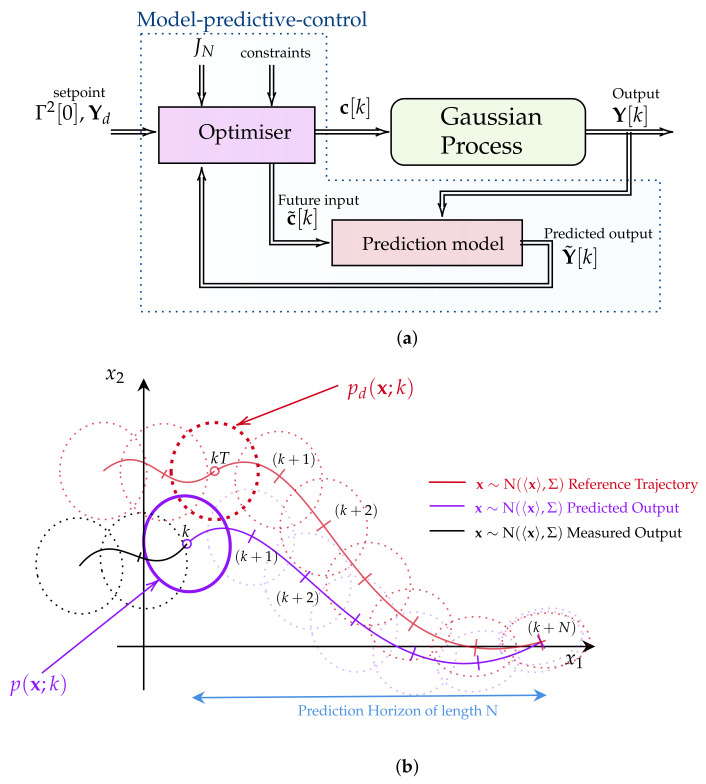
(**a**) A control diagram describing the main components of the implemented MPC methodology. The algorithm comprises a prediction model utilised to determine the optimal control c[k] using the interior point method with CasADi [44]. (**b**) A diagram illustrating a discrete MPC scheme applied to a second-order stochastic process. The algorithm predicts the behaviour of the dynamical system within a finite horizon of length *N* and compares it with the reference trajectory described by the PDF $p_{d}$.

For a clearer grasp of the MPC method’s application in solving the IL-QR problem in real-world scenarios, Figure 3a,b show the control diagram and the functionality of the MPC’s optimiser when considering a second-order stochastic system, respectively.

Figure 3a illustrates the real-time operation of the MPC algorithm. As the process evolves, the MPC algorithm receives input parameters, including a given set point $\Gamma^{2}\left[0\right],Y_{d}$, the prediction values $\tilde{Y}$ of μ and Σ from a prediction model, a set of constrains, the cost function $J_{N}$, and the current system dynamics Y, to solve the optimisation problem given in (13). Afterwards, the optimal control c solution of Equation (13) is applied to the system. The MPC method determines the optimal control c by utilising the differential equations of $\dot{\mu }$ and Σ as a prediction model in a finite horizon of length *N*. Figure 3b provides a brief overview of the working principle of the MPC’s optimiser block.

In the case of a stochastic process described by a bivariate time-varying PDF *p* with random variables $x_{1}$ and $x_{2}$, the MPC optimiser method initiates the optimisation process using the measured system’s PDF output (depicted in black). The optimisation process involves extrapolating the values of the PDF *p* within a finite horizon of length *N* and comparing them with the reference trajectory described by the PDF $p_{d}$. The optimisation problem is solved using the interior point method with CasADi [44]. In this study, the use of deterministic descriptions of the first two statistical moments over time has facilitated the control, prediction, and simulation of the PDF, owing to the nature of the Langevin equations under consideration. However, in scenarios involving pure data or more complex stochastic differential equations, estimating time-varying PDFs may require inference methods [62] or stochastic simulations [63].

## 4. Simulation Results

To demonstrate the numerical implementation of MPC for solving the IL-QR cost function (13), the subsequent subsections delve into applying the minimum information variability to both the Ornstein–Uhlenbeck process and the Kramers equation. Throughout the simulations, we utilise various parameters, which are conveniently summarised in Table 2. Each system undergoes a pair of simulations or “experiments”, as detailed in Table 2.

### 4.1. The O-U Process

**Figure 4:**
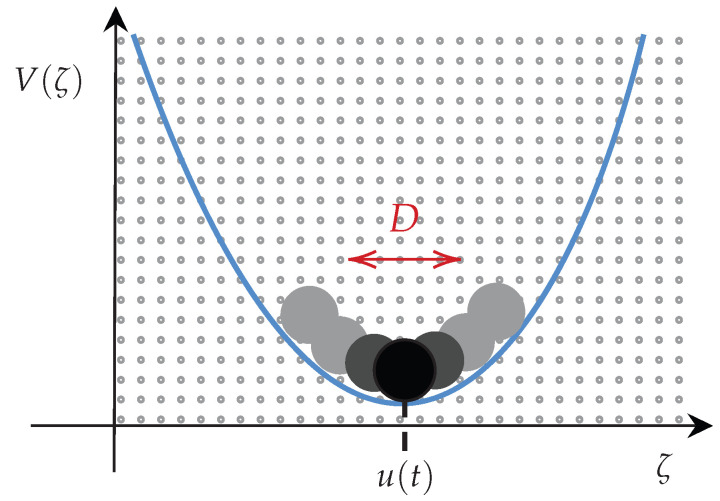
The O-U process equation is commonly used to describe a prototype of a noisy relaxation process, for instance, the movement of a particle confined to a harmonic potential $V\left(\zeta \right)=\frac{1}{2}\gamma {\left(\zeta -u\left(t\right)\right)}^{2}$ and thermal fluctuations with temperature *T* ($D=k_{B}T\gamma$, and $k_{B}$ is the Boltzmann constant) such that ζ(t) fluctuates stochastically.

First, let us consider the Ornstein–Uhlenbeck (O-U) process (see Figure 4) defined by the following Langevin equation:

(17) $$\dot{\zeta }=-\gamma \left(\zeta \left(t\right)-u\left(t\right)\right)+\xi \left(t\right),$$

where ζ(t) is a random variable, u(t) is a deterministic force, γ is a damping constant, and ξ(t) is a short correlated random forcing such that $\langle \xi \left(t\right)\xi \left(t_{1}\right)\rangle =2D\delta \left(t-t_{1}\right)$ with D≥0 and 〈ξ(t)〉=0.

**Figure 5:**
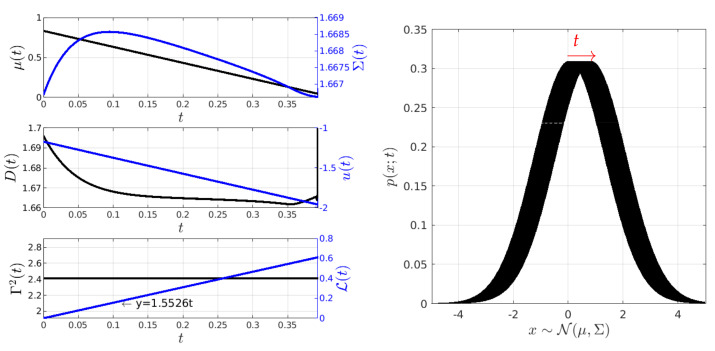
Minimum information variability control of the O-U process (Experiment 1). In this experiment, the initial and desired PDFs have similar variances but different mean values. In the simulation, $\Gamma^{2}$ is maintained constant at a value of $\Gamma^{2}=2.4$. To follow the geodesic, the input force u(t) needs to linearly decrease, while the noise amplitude D(t) decreases slightly exponentially. Conversely, the mean value undergoes a linear change, while the variance exhibits hyperbolic behaviour.

**Figure 6:**
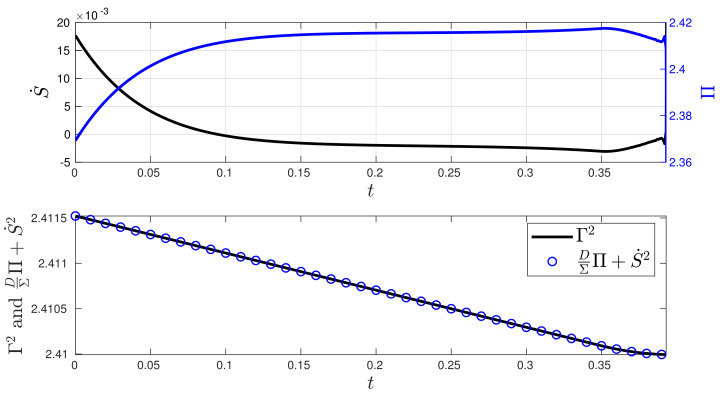
Stochastic thermodynamics under minimum information variability for the O-U process (Experiment 1). The second plot demonstrates that the balance expressed via Equation (9) is maintained as expected. The control generates a small entropy rate $\dot{S}$ converging to a negative value, indicating that most of the entropy flows from the system to the environment. The control induces entropy production to maintain the system out of equilibrium with respect to $Y_{d}$. This departure from equilibrium is supported by the presence of entropy production Π>0.

**Figure 7:**
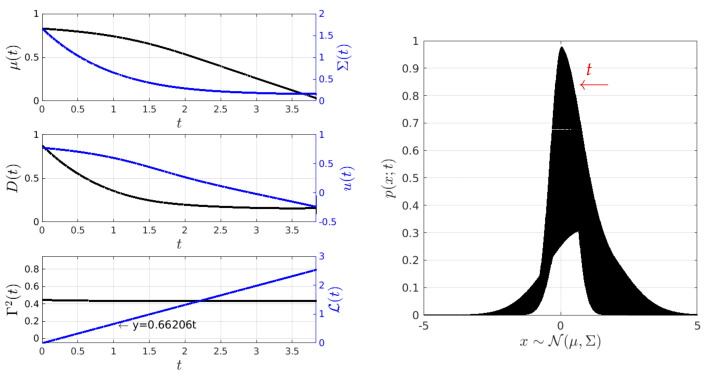
Minimum information variability control of the O-U process (Experiment 2). In this experiment, the initial and desired PDFs have different variances and mean values. $\Gamma^{2}$ is maintained constant at a value of $\Gamma^{2}=0.41$, while both the input force u(t) and the noise amplitude D(t) decrease exponentially.

**Figure 8:**
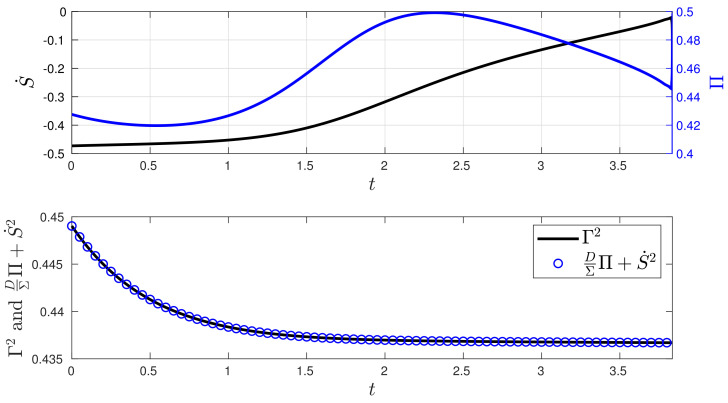
Stochastic thermodynamics under minimum information variability for the O-U process (Experiment 2). The plot of Equation (9) demonstrates that to maintain a constant information rate Γ while being out of equilibrium, we increase the value of entropy production Π, and most of the entropy flows out to the environment, as indicated by the negative sign of $\dot{S}$.

The results of the MPC implementation are shown in Figure 5, Figure 6, Figure 7 and Figure 8. In the following, it is important to note that, in the figures, black colour is used to represent values labelled on the y-left axis, while blue colour is used for values labelled on the y-right axis, unless otherwise specified. Figure 5 illustrates the scenario where the desired state $Y_{d}$ of the O-U process is $Y_{d}={\left[1/30,1/\left(2\times 0.3\right)\right]}^{⊤}$. It also shows the time evolution of the states Y(t)=[μ,β(t)] and controls $c\left(t\right)={\left[u\left(t\right),D\left(t\right)\right]}^{⊤}$. From the results, we see that the method finds a geodesic motion (solution to the IL-QR) from the initial to the final state in less than 0.4 seconds. The geodesic motion is corroborated by the constant value of $\Gamma^{2}\left(t\right)\approx \Gamma^{2}\left(0\right)=2.4$ and the plot of the information length L whose shape is a line with a slope of 1.5526. $\Gamma^{2}\left(0\right)$ is computed by considering that u(t=0)=D(t=0)=0. It is noteworthy that the value of Σ is observed to vary slightly over time compared to the hyperbolic analytical solution in [4], which is provided for a non-constant Σ (refer to Appendix A).

When analysing the stochastic thermodynamics of the O-U system controlled by the MPC technique, Figure 6 presents the plot of the entropy rate $\dot{S}$ in comparison with the entropy production Π, along with a plot of the value of $\Gamma^{2}$ using Equation (9). The analytical expressions for $\dot{S}$ and Π, along with their derivations, are given in Appendix C. In the closed-loop system, we can see that the MPC method induces slight changes in both the entropy production Π and the entropy rate $\dot{S}$ during the process. Since the values of Σ and μ in the desired state $Y_{d}$ are close to, and far from, their initial conditions at state Y(0), respectively, the balance between $\dot{S},\Pi$, and $\Gamma^{2}$ as given by (9) is kept by maintaining an almost constant D(t) and a u(t) with a nearly constant velocity.

Under conditions almost similar to those in Figure 5, Figure 7 shows the behaviour of the closed-loop system PDF, the states μ and Σ, and the controls *D* and *u*, as well as the behaviour of $\Gamma^{2}$ for $Y_{d}\left(t\right)={\left[1/30,1/\left(2\times 3\right)\right]}^{⊤}$. Here, $\Gamma^{2}\left(0\right)$ is computed by considering that u(t=0)=0 and D(t=0)=1/(2×0.3). The final state $Y_{d}$ is achieved at approximately t=2.8. Once again, the geodesic behaviour is supported by the constant value of $\Gamma^{2}\left(t\right)\approx \Gamma^{2}\left(0\right)=0.41$ and the plot of the information length L, which depicts a line with a slope of 0.64759.

In comparison to the stochastic thermodynamics shown in Figure 6, Figure 8 exhibits minor changes in the entropy production Π and significant variations in the entropy rate $\dot{S}$ of the closed-loop system, given that the values of Σ and μ in the desired state $Y_{d}$ differ from its initial condition Y(0). This disparity also leads to slight fluctuations in both the D(t) and u(t). Additionally, Figure 8 demonstrates the validity of the balance in Equation (9).

### 4.2. Kramers Equation

To study the solution of the IL-QR problem in a higher-order system via the MPC method, let us consider the so-called Kramers equation. The Kramers equation describes the Brownian motion of particles in an external field [12]. The non-autonomous version of the Kramers equation is given by the following set of Langevin equations:

(18) $$\begin{array}{c} \left[\begin{array}{c} {\dot{\zeta }}_{1}\\ {\dot{\zeta }}_{2} \end{array}\right]=\left[\begin{array}{cc} 0 & 1\\ -\omega^{2} & -\gamma \end{array}\right]\left[\begin{array}{c} \zeta_{1}\\ \zeta_{2} \end{array}\right]+\left[\begin{array}{c} 0\\ 1 \end{array}\right]\xi \left(t\right)+\left[\begin{array}{c} 0\\ 1 \end{array}\right]u\left(t\right) \end{array}$$

Here, ξ is a short correlated Gaussian noise with a zero mean 〈ξ〉=0 and a strength D≥0 with the following property:

(19) $$\langle \xi \left(t\right)\xi \left(t^{\prime }\right)\rangle =2D\delta \left(t-t^{\prime }\right).$$

**Figure 9:**
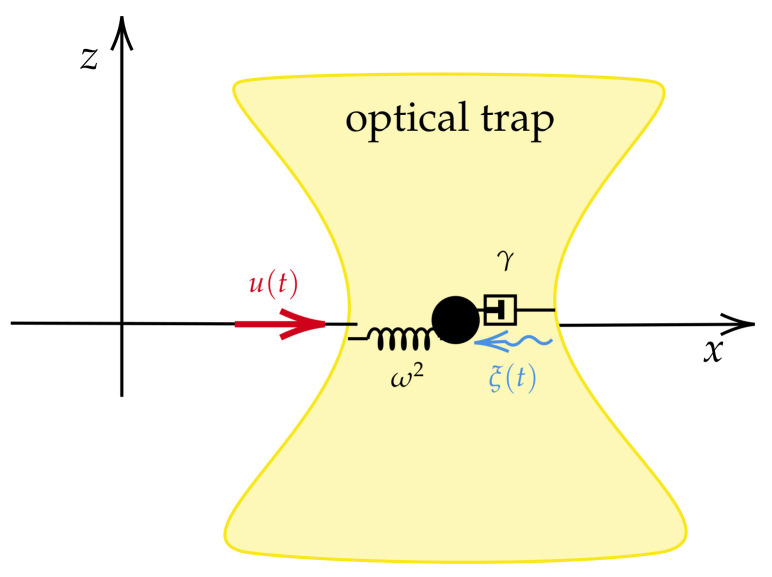
Mechanical representation of the Kramers Equation (18) as a mass–spring–damper system. In this system, the external force u(t) is deterministic, while ξ(t) represents a stochastic perturbation on the process, which varies due to the temperature of the fluid [12].

In practice, as shown in Figure 9, the Kramers Equation (18) is also a good first approximation to describe the dynamics in one-dimension of a particle in an optical trap [5]. The Kramers equation control c and state Y vectors are defined by

(20) $$\begin{array}{ccc} c & = & {\left[u,D\right]}^{⊤}, \end{array}$$

(21) $$\begin{array}{ccc} Y & = & {\left[\mu_{1},\mu_{2},\Sigma_{11},\Sigma_{12},\Sigma_{22}\right]}^{⊤}. \end{array}$$

Here, $\mu_{1}$ and $\mu_{1}$ are the mean values of the random variables $\zeta_{1}$ and $\zeta_{2}$, respectively. $\Sigma_{11}$, $\Sigma_{12}$, and $\Sigma_{22}$ are the values describing the covariance matrix Σ.

**Figure 10:**
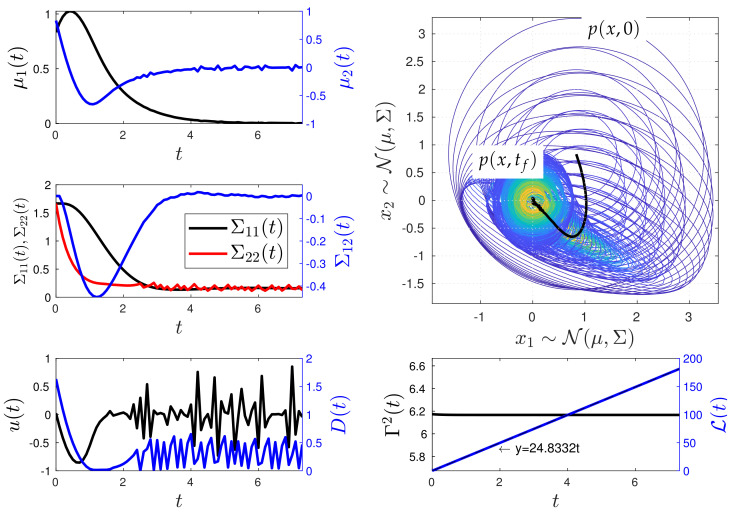
Minimum information variability control of the Kramers equation (Experiment 1). The control effectively adjusts the values of the covariance matrix Σ and the mean vector μ by dynamically modifying *u* and *D*, transitioning the system’s PDF from one state to another out of equilibrium. It is noteworthy that, to maintain the system on the IL’s geodesic, the MPC method maintains a constant $\Gamma^{2}$, resulting in abrupt changes in *u* and *D* as the system approaches the desired state $Y_{d}$. Physically, this leads to a high entropy production due to the control action.

**Figure 11:**
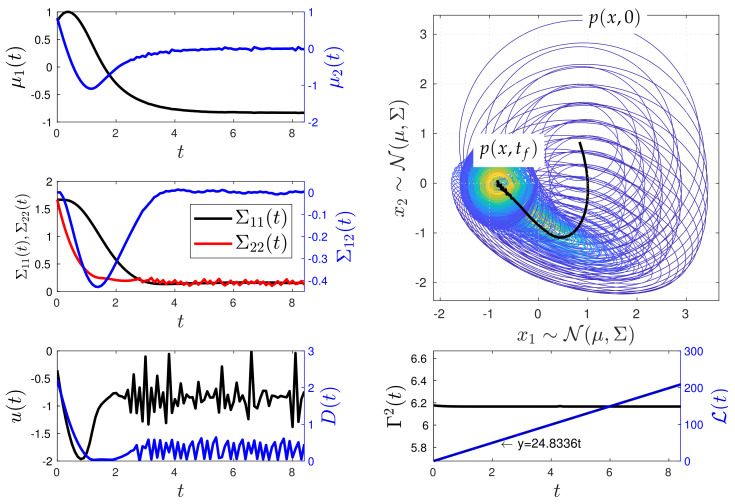
Minimum information variability control of the Kramers equation (Experiment 2). In this scenario, the value of $Y_{d}$ results in slower dynamics compared to Experiment 1, as MPC now requires more time to reach $Y_{d}$. Once again, significant changes are observed in *u* and *D* to maintain a constant information rate Γ when the system approaches the desired state $Y_{d}$.

Figure 10 and Figure 11 show the simulation results of the closed-loop Kramers equation when considering the desired states $Y_{d}\left(t\right)={\left[0,0,1/\left(2\times 3\right),0,1/\left(2\times 3\right)\right]}^{⊤}$ and $Y_{d}\left(t\right)={\left[-5/6,0,1/\left(2\times 3\right),0,1/\left(2\times 3\right)\right]}^{⊤}$, respectively. These figures include the time evolution plots of the mean values $\mu_{1}$ and $\mu_{2}$ and the covariance matrix values $\Sigma_{11}$, $\Sigma_{12}$, and $\Sigma_{22}$ of the Kramers equation random variables $\zeta_{1}$ and $\zeta_{2}$. In addition, they include the time evolution of the bivariate PDF p(x;t) with the spatial vector $x={\left[x_{1},x_{2}\right]}^{⊤}$, with $x_{1}$ and $x_{2}$ representing the position and velocity of the system dynamics, respectively. The remaining parameters used in the simulations are listed in Table 2. Again, in the figures, black colour is used to represent values labelled on the y-left axis, while blue colour is used for values labelled on the y-right axis, unless otherwise specified.

For the first numerical experiment, Figure 10 demonstrates that the MPC method is capable of maintaining $\Gamma^{2}$ as constant through time while reaching the desired state $Y_{d}$. Here, the value of Γ(0) in (13) is obtained as follows:

(22) $$\begin{array}{c} \Gamma^{2}\left(0\right)={\left(A\mu (0)+Bu(0)\right)}^{⊤}\Sigma^{-1}\left(0\right)\left(A\mu (0)+Bu(0)\right)\\ \;\;\;\;\;\;\;\;+\frac{1}{2}\mathrm{Tr}\left({\left(\Sigma^{-1}\left(0\right)\left(A\Sigma \left(0\right)+\Sigma \left(0\right)A^{⊤}+2D\left(0\right)\right)\right)}^{2}\right)=6.16667, \end{array}$$

where u(0)=0 and $\mathrm{vec}\left(D\left(0\right)\right)={\left[0,0,0,1/\left(2\times 0.3\right)\right]}^{⊤}$, while μ(0),Σ(0) and A are taken from the corresponding Y(0) and the mathematical model (18), respectively. The geodesic dynamics give a behaviour with slow oscillations in the state Y. The controls *u* and *D* present high oscillations as the system reaches the desired state $Y_{d}$. The system reaches $Y_{d}$ at t≈7 with an error of $1\times 10^{-3}$. The geodesic behaviour is supported by the linear behaviour of the information length L compared to the fitted equation y=24.8332t.

Figure 10 shows a second numerical experiment where $Y_{d}$ is even farther from the system’s equilibrium. Yet, the MPC method can maintain $\Gamma^{2}$ as constant through time while reaching $Y_{d}$. Like the example of Figure 10, in this case $\Gamma^{2}\left(0\right)=6.16667$. Small oscillations persists in the time evolution of $\mu_{1}$, $\mu_{2}$, $\Sigma_{11}$, c$\Sigma_{12}$, and $\Sigma_{22}$. The system reaches the desired state $Y_{d}$ at t≈8.5. Thus, the farther the desired state $Y_{d}$ is from the initial state Y, the longer the time it takes to reach it while following the geodesic path. The geodesic behaviour is evidenced by the plot of the information length L, whose behaviour is compared to the fitted equation y=24.8336t. Similar to the example in Figure 10, the controls exhibit highly oscillatory behaviour as the system reaches $Y_{d}$.

## 5. Conclusions

In this work, we demonstrated the application of the MPC method, an online optimisation algorithm for constrained control problems, to achieve the minimum information variability in systems governed by linear stochastic differential equations. The linearity of the system results in time-varying Gaussian PDFs, with statistical moments determined by a set of deterministic differential equations. Our simulations demonstrate that the MPC method is a practical approach for determining the geodesic of the information length between the initial and desired probabilistic states through the solution of the proposed IL-QR loss function.

From a thermodynamic standpoint, simulations of the MPC in the O-U process reveal that the MPC directly influences the amount of entropy production generated by the system to meet all optimisation requirements. Future work will involve extending this approach to nonlinear stochastic dynamics, such as toy models [64], systems with uncertain physical parameters [65], Brownian motion [66], or diffusion [67,68]. This extension aims to maximise the free energy [21] by minimising the entropy production and to analyse closed-loop stochastic thermodynamics for higher-order systems. It is worth noting that extending the method to nonlinear dynamics can be achieved through the Laplace assumption [69] and/or by employing Kalman/particle filter methods [70,71]. 

## Figures and Tables

**Table 1:** Description of the different variables used throughout this work.

Symbol	Description
ζ	Random vector variable
x	Spatial variable of the PDF
ξ	Gaussian stochastic variable
u(t)	Bounded smooth external input (any time-dependent function)
D(t)	Time-dependent noise amplitude matrix
μ	Mean value vector of the random vector ζ
Σ	Covariance matrix of the random vector ζ
p(x;t)	Probability density function (PDF)
L(t)	Information length
Γ(t)	Information rate
$\dot{S}$	Entropy rate
Π	Entropy production
Φ	Entropy flow
Y(t)	Vector state composed by the elements of μ and Σ at time *t*. The vector describes the current PDF at time *t*.
$Y_{d}$	Desired vector state. The vector describes the desired Gaussian PDF.
c(t)	Vector of controls including *u* and the elements $D_{ij}$ of the amplitude noise matrix D
Q	Weight matrix regulating the error between Y(t) and $Y_{d}$
R	Weight matrix regulating the control action
$I_{L}$	Weight factor of the error between the current information rate Γ(t) at time *t* and the initial information rate Γ(0) to keep it constant at all *t*
$\hat{\Gamma }$	Predicted information rate. The ^ symbol implies prediction.
μ[k]	Discrete time mean value vector. The brackets [k], where k=0,1,2,…, refers to the discrete time sampled at time period $T_{s}$.
γ	Damping constant
ω	Undamped natural frequency constant
$T_{s}$	Sampling period
*N*	Prediction horizon length

**Table 2:** Parameters used in the simulation results for the O-U and Kramers systems. The table includes figure numbers showing the simulation where the set of parameters was used.

System	Experiment	Figure	Y(0)			Y_*d*_(t)			$\Gamma^{2}$(0)
O-U	1	5, 6	${\left[5/6,1/\left(2\times 0.3\right)\right]}^{⊤}$			${\left[1/30,1/\left(2\times 0.3\right)\right]}^{⊤}$			2.4
	2	7, 8	${\left[5/6,1/\left(2\times 0.3\right)\right]}^{⊤}$			${\left[1/30,1/\left(2\times 3\right)\right]}^{⊤}$			0.41
Kramers	1	10	${\left[5/6,5/6,1/\left(2\times 0.3\right),0,1/\left(2\times 0.3\right)\right]}^{⊤}$			${\left[0,0,1/\left(2\times 3\right),0,1/\left(2\times 3\right)\right]}^{⊤}$			6.16667
	2	11	${\left[5/6,5/6,1/\left(2\times 0.3\right),0,1/\left(2\times 0.3\right)\right]}^{⊤}$			${\left[-5/6,0,1/\left(2\times 3\right),0,1/\left(2\times 3\right)\right]}^{⊤}$			6.16667
**System**	**Experiment**	**Figure**	***γ***	***ω***	***T_s_***	***N***	***I_L_***	**R**	**Q**
O-U	1	5, 6	1	-	$1\times 10^{-3}$	50	$1\times 10^{3}$	$1\times 10^{-2}I_{2}$	$Q_{12}=Q_{21}=0,Q_{11}=1\times 10^{2}$ and $Q_{22}=5\times 10^{2}$
	2	7, 8	1	-	$1\times 10^{-3}$	50	$1\times 10^{4}$	$1\times 10^{-2}I_{2}$	$Q_{12}=Q_{21}=0,Q_{11}=1\times 10^{2}$ and $Q_{22}=5\times 10^{2}$
Kramers	1	10	2	1	$1\times 10^{-1}$	50	$5\times 10^{3}$	$1\times 10^{-5}I_{3}$	$1\times 10^{2}I_{5}$
	2	11	2	1	$1\times 10^{-1}$	50	$5\times 10^{3}$	$1\times 10^{-5}I_{3}$	$1\times 10^{2}I_{5}$

**Figure A1:**
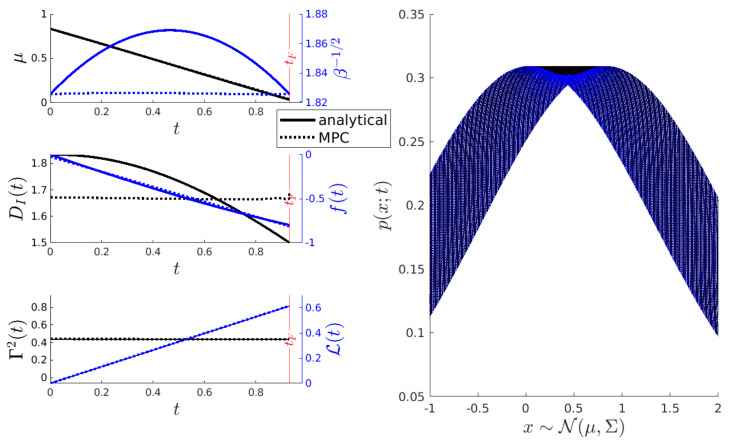
Comparison between the analytical solution of Equations (A3) and (A4) vs. the IL-QR solution for the O-U process (17). The IL-QR parameters are $Y\left(0\right)={\left[5/6,1/\left(2\times 0.3\right)\right]}^{⊤}$, $Y_{d}\left(t\right)={\left[1/30,1/\left(2\times 0.3\right)\right]}^{⊤}$, γ=1, $T_{s}=1\times 10^{-3}$, N=50, $I_{L}=1\times 10^{3}$, $R=1\times 10^{-4}I_{2}$, and $Q=1\times 10^{2}I_{2}$.

**Figure A2:**
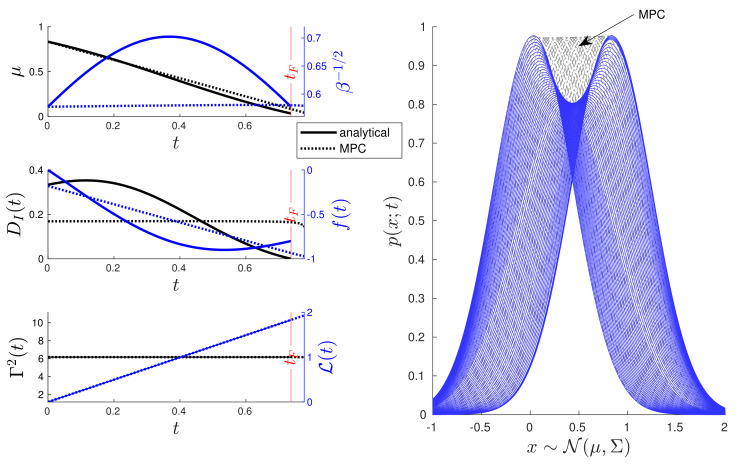
Comparison of the analytical solution of Equations (A3) and (A4) vs. the IL-QR solution for the O-U process (17). The IL-QR parameters are $Y\left(0\right)={\left[5/6,1/\left(2\times 3\right)\right]}^{⊤}$, $Y_{d}\left(t\right)={\left[1/30,1/\left(2\times 3\right)\right]}^{⊤}$, γ=1, $T_{s}=1\times 10^{-3}$, N=10, $I_{L}=1\times 10^{3}$, $R=1\times 10^{-4}I_{2}$, and $Q=1\times 10^{2}I_{2}$.

## Data Availability

No new data were created or analyzed in this study. Data sharing is not applicable to this article.

## Abbreviations

The following abbreviations are used in this manuscript:

PDFs	Probability density functions
FP	Fokker–Planck
IL	Information length
MPC	Model predictive control
QR	Quadratic regulator
BIBO	Bounded-input, bounded-output

## Appendix A. A Solution by the Euler–Lagrange Equation

In [4], an analytical solution describing the geodesic motion connecting given initial p(x;0) and final $p(x;t_{F})$ probability distributions is found by solving the Euler–Lagrange equations in terms of the covariance Σ and mean μ value of a first-order stochastic process. Here, we take the O-U process to compare such an analytical solution of the geodesic dynamics with the solution obtained by the MPC method. Additionally, we provide the set of differential equations describing the geodesic motion for an *n*-th order Gaussian process.

The Euler–Lagrange equations for the Lagrangian $\Gamma^{2}$ in terms of the vector μ (mean value) and the matrix Σ (covariance) are

(A1) $$\begin{array}{ccc} \frac{d}{dt}\left(\frac{\partial \Gamma^{2}\left(t\right)}{\partial \dot{\mu }}\right) & = & \frac{\partial \Gamma^{2}\left(t\right)}{\partial \mu }, \end{array}$$

(A2) $$\begin{array}{ccc} \frac{d}{dt}\left(\frac{\partial \Gamma^{2}\left(t\right)}{\partial \dot{\Sigma }}\right) & = & \frac{\partial \Gamma^{2}\left(t\right)}{\partial \Sigma }. \end{array}$$

Using (7) in (A1) and (A2), we obtain (see Appendix B)

(A3) $$\begin{array}{ccc} \ddot{\mu }+\dot{\Sigma }\Sigma^{-1}\dot{\mu } & = & 0, \end{array}$$

(A4) $$\begin{array}{ccc} \ddot{\Sigma }+\dot{\mu }{\dot{\mu }}^{⊤}-\dot{\Sigma }\Sigma^{-1}\dot{\Sigma } & = & 0. \end{array}$$

As mentioned above, [4] presents a closed-form analytical solution to the boundary value problem of Equations (A3) and (A4) when the dimensions of both μ and Σ are one. Specifically, Equations (A3) and (A4) have a trivial solution where Σ is constant. For a non-constant Σ, the following hyperbolic solutions are found in [4]:

(A5) $$\begin{array}{ccc} \beta (t) & = & \beta_{*}\cosh\left[\frac{1}{2}\sqrt{\alpha }\left(t-A\right)\right], \end{array}$$

(A6) $$\begin{array}{ccc} \mu (t) & = & -\frac{1}{\sqrt{\beta_{*}}}\tanh\left[\frac{1}{2}\sqrt{\alpha }\left(t-A\right)\right]+\mu_{M}. \end{array}$$

Here, $\beta =\frac{1}{2\Sigma }$ and $\mu \left(t=A\right)=\left(\mu \left(0\right)+\mu \left(t_{F}\right)\right)/2=\mu_{M}$. The values of $\beta_{*},\alpha$, and *A* are computed using a given set of boundary conditions (for a complete discussion, see [4]). For instance, the parameter

(A7) $$A=\frac{1}{\gamma \sqrt{\beta (0)}\mu \left(0\right)}\frac{Q}{\cosh(Q)},$$

where $Q=\sinh^{-1}\left(\frac{\phi }{2}\right)$ using $\phi =\sqrt{\beta (0)}\left(\mu \left(0\right)-\mu \left(t_{F}\right)\right)$. Clearly, through Equations (A5) and (A6) and the dynamical model of the O-U process (17), we can construct the optimal control f(t) of the input u(t) and the optimal noise amplitude $D_{I}\left(t\right)$ of D(t). From [4], given that u(0)=0, such optimal controls are given by

(A8) $$\begin{array}{ccc} f(t) & = & \mu \left(t\right)-\frac{\beta (0)\mu (0)}{\beta (t)}, \end{array}$$

(A9) $$\begin{array}{ccc} D_{I}\left(t\right) & = & \frac{1}{2\beta (t)}\left[\gamma -\frac{\sqrt{\alpha }}{2}\tanh\left(\frac{1}{2}\sqrt{\alpha }\left(t-A\right)\right)\right]. \end{array}$$

To compare the analytical and MPC solutions of the geodesic of the IL, Figure A1 shows the behaviour of the O-U process when controlled through the analytical solutions (A8) and (A9) or the IL-MPC method. The figure contains different subplots that show the time evolution of μ, $\beta^{1/2}$, $\Gamma^{2}$, and L and the optimal controls $D_{I}$ and *f*. In the simulation, the desired state and the damping are $Y_{d}\left(t\right)={\left[1/30,1/\left(2\times 0.3\right)\right]}^{⊤}$ and γ=1, respectively. Additionally, we set a fixed final time $t_{F}=2A=0.9304$ (one cycle of the hyperbolic geodesic motion (A5) and (A6)) by considering the initial state $Y\left(0\right)={\left[5/6,1/\left(2\times 0.3\right)\right]}^{⊤}$. Figure A1 uses dashed and non-dashed lines for the MPC and the analytical response, respectively. Additionally, recall that black colour is used to represent values labelled on the y-left axis, while blue colour is used for values labelled on the y-right axis. From the comparison, a major conclusion is that the time evolution of β is no longer hyperbolic when using the MPC method. This means that the MPC method finds an almost constant Σ solution but not the hyperbolic solution shown in [4]. The MPC allows us to reach the final state $Y_{d}$ at $t_{F}$ with an error of $6.6\times 10^{-4}$.

As a second example, Figure A2 shows the dynamics of the controlled O-U process when the initial state is $Y\left(0\right)={\left[5/6,1/\left(2\times 3\right)\right]}^{⊤}$ (fixing $t_{F}=2A=0.7367$), the desired state $Y_{d}\left(t\right)={\left[1/30,1/\left(2\times 3\right)\right]}^{⊤}$, and the damping γ=1. Again, the MPC method recovers a geodesic solution where the β time evolution is constant. In this scenario, the MPC method reaches the desired state $Y_{d}$ with an error of $9.8\times 10^{-4}$ in a time $t>t_{F}$, demonstrating that the numerical optimisation scheme may not recover an optimal time.

As a final remark, note that if the *n*-th order case is considered, Equations (A3) and (A4) form a set of non-linear differential equations whose solution may be obtained by a numerical procedure. But, even for the case of a second-order stochastic process, this becomes a challenging problem (we have a boundary value problem of 12 non-linear differential equations). Hence, the MPC method provides an alternative solution to this problem while being an experimentally feasible approach, as demonstrated by the application to the Kramers equation in Section 4.2.

## Appendix B. Geodesic Dynamics Derivation

Based on matrix calculus identities from [72], we can derive the Euler–Lagrange equations for $\Gamma^{2}\left(t\right)$. First, for μ we have

(A10) $$\begin{array}{c} \frac{d}{dt}\left(\frac{\partial \Gamma^{2}\left(t\right)}{\partial \dot{\mu }}\right)=0, \end{array}$$

where $\frac{\partial \Gamma^{2}\left(t\right)}{\partial \dot{\mu }}=2\Sigma^{-1}\dot{\mu }$. Therefore,

(A11) $$\frac{d}{dt}\left(\Sigma^{-1}\right)\dot{\mu }+\Sigma^{-1}\ddot{\mu }=-\Sigma^{-1}\dot{\Sigma }\Sigma^{-1}\dot{\mu }+\Sigma^{-1}\ddot{\mu }=0,$$

which leads to Equation (A3). For Σ, we have

(A12) $$\begin{array}{ccc} \frac{\partial \Gamma^{2}\left(t\right)}{\partial \dot{\Sigma }} & = & \frac{1}{2}\mathrm{Tr}\left(\Sigma^{-1}\left(\partial \dot{\Sigma }\right)\Sigma^{-1}\dot{\Sigma }+\Sigma^{-1}\dot{\Sigma }\Sigma^{-1}\left(\partial \dot{\Sigma }\right)\right)\\ & = & \frac{1}{2}\left(2{\left(\Sigma^{-1}\dot{\Sigma }\Sigma^{-1}\right)}^{⊤}\right), \end{array}$$

(A13) $$\begin{array}{ccc} \frac{d}{dt}\left(\frac{\partial \Gamma^{2}\left(t\right)}{\partial \dot{\Sigma }}\right) & = & \frac{d}{dt}\left(\Sigma^{-1}\dot{\Sigma }\Sigma^{-1}\right)\\ & = & -\Sigma^{-1}\dot{\Sigma }\Sigma^{-1}\dot{\Sigma }\Sigma^{-1}+\Sigma^{-1}\ddot{\Sigma }\Sigma^{-1}\\ & & -\Sigma^{-1}\dot{\Sigma }\Sigma^{-1}\dot{\Sigma }\Sigma^{-1}, \end{array}$$

$$\begin{array}{ccc} \frac{\partial \Gamma^{2}\left(t\right)}{\partial \Sigma } & = & -\Sigma^{-1}\dot{\mu }{\dot{\mu }}^{⊤}\Sigma^{-1}+\frac{1}{2}\frac{\partial }{\partial \Sigma }\mathrm{Tr}\left(\Sigma^{-1}\dot{\Sigma }\Sigma^{-1}\dot{\Sigma }\right)\\ & = & -\Sigma^{-1}\dot{\mu }{\dot{\mu }}^{⊤}\Sigma^{-1}+\frac{1}{2}\mathrm{Tr}\left(\left(\partial \Sigma^{-1}\right)\dot{\Sigma }\Sigma^{-1}\dot{\Sigma }\right.\\ & & \left.+\Sigma^{-1}\dot{\Sigma }\left(\partial \Sigma^{-1}\right)\dot{\Sigma }\right) \end{array}$$

(A14) $$\begin{array}{ccc} & = & -\Sigma^{-1}\dot{\mu }{\dot{\mu }}^{⊤}\Sigma^{-1}+\frac{1}{2}\left(-2{\left(\Sigma^{-1}\dot{\Sigma }\Sigma^{-1}\dot{\Sigma }\Sigma^{-1}\right)}^{⊤}\right). \end{array}$$

## Appendix C. Entropy Rate in the O-U Process

The O-U process Fokker–Planck equation is given by

(A15) $$\frac{\partial }{\partial t}p\left(x,t\right)=-\frac{\partial }{\partial x}\left[f(x,t)p(x,t)\right]+D\frac{\partial^{2}}{\partial x^{2}}p\left(x,t\right)=-\frac{\partial }{\partial x}J,$$

where f(x,t)=−γ(x+u(t)) and $J=fp-D\partial_{x}p$. The solution of (A15) is Gaussian and given by

(A16) $$p\left(x,t\right)=\frac{1}{\sqrt{2\pi \Sigma }}e^{-\frac{{\left(x-\mu \right)}^{2}}{2\Sigma }}.$$

The values of the entropy production Π and entropy flow Φ for the O-U process can be obtained from the computation of the entropy rate of the solution p(x,t) of the Fokker–Planck Equation (A15) as follows (see also [21,50]):

(A17) $$\begin{array}{ccc} \dot{S} & = & -\int \;dx\;\dot{p}\left(x,t\right)\ln p(x,t)\\ & = & -\int \;dx\left(-\partial_{x}J\left(x,t\right)\right)\ln p(x,t)\\ & = & -\int \;dxJ\left(x,t\right)\partial_{x}\ln p(x,t)\\ & = & -\int \;dxJ\left(x,t\right)\frac{1}{p(x,t)}\left(\frac{f(x,t)p(x,t)-J(x,t)}{D}\right)\\ & = & \int \;dx\frac{{\left(J\left(x,t\right)\right)}^{2}}{Dp(x,t)}-\int \;dx\frac{J(x,t)f(x,t)}{D}\\ & = & \Pi -\Phi . \end{array}$$

Hence, the exact values of Π and $\dot{S}$ are [21]

(A18) $$\begin{array}{ccc} \dot{S} & = & \frac{1}{2}\frac{\dot{\Sigma }}{\Sigma }, \end{array}$$

(A19) $$\begin{array}{ccc} \Pi & = & \frac{\dot{\mu }}{D}+\frac{\gamma^{2}\Sigma }{D}+\frac{D}{\Sigma }-2\gamma . \end{array}$$
